# Antibody Binding Selectivity: Alternative Sets of Antigen Residues Entail High-Affinity Recognition

**DOI:** 10.1371/journal.pone.0143374

**Published:** 2015-12-02

**Authors:** Yves Nominé, Laurence Choulier, Gilles Travé, Thierry Vernet, Danièle Altschuh

**Affiliations:** 1 Equipe labellisée Ligue 2015—UMR 7242, CNRS—Université de Strasbourg, CNRS, Illkirch, France; 2 Université Grenoble Alpes, IBS, Grenoble, France; 3 CNRS, IBS, Grenoble, France; 4 CEA, IBS, Grenoble, France; Bioinformatics Institute, SINGAPORE

## Abstract

Understanding the relationship between protein sequence and molecular recognition selectivity remains a major challenge. The antibody fragment scFv1F4 recognizes with sub nM affinity a decapeptide (sequence ^6^TAMFQDPQER^15^) derived from the N-terminal end of human papilloma virus E6 oncoprotein. Using this decapeptide as antigen, we had previously shown that only the wild type amino-acid or conservative replacements were allowed at positions 9 to 12 and 15 of the peptide, indicating a strong binding selectivity. Nevertheless phenylalanine (F) was equally well tolerated as the wild type glutamine (Q) at position 13, while all other amino acids led to weaker scFv binding. The interfaces of complexes involving either Q or F are expected to diverge, due to the different physico-chemistry of these residues. This would imply that high-affinity binding can be achieved through distinct interfacial geometries. In order to investigate this point, we disrupted the scFv–peptide interface by modifying one or several peptide positions. We then analyzed the effect on binding of amino acid changes at the remaining positions, an altered susceptibility being indicative of an altered role in complex formation. The 23 starting variants analyzed contained replacements whose effects on scFv1F4 binding ranged from minor to drastic. A permutation analysis (effect of replacing each peptide position by all other amino acids except cysteine) was carried out on the 23 variants using the PEPperCHIP® Platform technology. A comparison of their permutation patterns with that of the wild type peptide indicated that starting replacements at position 11, 12 or 13 modified the tolerance to amino-acid changes at the other two positions. The interdependence between the three positions was confirmed by SPR (Biacore® technology). Our data demonstrate that binding selectivity does not preclude the existence of alternative high-affinity recognition modes.

## Introduction

The determinants of binding selectivity in protein-protein interactions remain largely unexplored even though their knowledge is crucial for understanding binding events that underlie biological phenomena or for developing new drugs. Selectivity is the ability of a molecule to discriminate between interaction partners. A selective binder shows little cross-reactivity: it recognizes a given partner with much higher affinity than other partners. A non-selective binder is highly cross-reactive: it recognizes a range of molecules with similar affinities. The description of selectivity is necessarily operational since it depends on the number and nature of molecules analyzed. Ideally it should be based on the quantitative characterization of a very large number of interactions, but this task is seldom expanded beyond a few dozen, typically alanine variants of interfacial residues.

Alanine scanning mutagenesis (Ala-scan) experiments have shown that a small proportion of all residues that compose protein interfaces play a major role in binding [1–2]. They were called hot spots and defined as residues whose Ala replacement decreases the binding free energy (ΔΔG) by more than 2 kcal/mol. Hot spots are generally clustered at the center of the binding site and are surrounded by energetically less important residues, initially proposed to shield hot spots from the solvent (O-ring hypothesis [2]). Hot spots are enriched in Trp, Tyr and Arg, which was attributed to the capacity of these amino-acids for multiple interaction types (aromatic-π, h-bond, hydrophobic) [2–3]. Amino-acid preferences at binding sites were also investigated using structural data for protein complexes. The conclusions differ somewhat depending on the data set and definition of interface residues, but a preferential contribution to binding sites of hydrophobic, aromatic and Arg residues was observed [4–7]. These amino-acids were proposed to be well suited for making contacts because of stickiness, flexibility and mixed physico-chemical properties, allowing them to interact with different residues via different contact types. It was suggested that hydrophobic residues (generally identified as hot spots and located at the center of the binding site) mainly provide affinity, while surrounding polar residues contribute to specificity [6, 8–9].

Beyond the identification of general rules, the compilation of Ala-scan experiments and the analysis of binding site architectures stress the complex relationship between sequence/structure and binding [10–12]. Furthermore the hypothesis that hot spot residues present some recognition adaptability raises the question of the basis of selectivity. Statistical studies must therefore be complemented with in-depth studies of the relation between structure and binding in individual interactions. Because experimental mutational studies are laborious and time-consuming, much effort was invested in the computational prediction of residue contributions to binding energy from the 3D structure of complexes [13–16]. While reasonable success was achieved in the identification of hot spots, precise prediction of the variation in binding energy from *in silico* Ala-scan remains problematic. Predicting the effect on binding energy of replacements other than Ala or of multiple replacements, which is crucial for a description of selectivity, is even more elusive. One of the reasons for the moderate success of quantitative predictions is that protein plasticity and dynamics play a significant role in binding [17–20]. Differences between free and bound molecules include structural re-organizations, changes in internal dynamics, ordering of mobile regions and desolvation effects, with complex consequences on enthalpy and entropy variations. The description of these phenomena, of their influence on binding energy, and of the way they change upon interface modifications, is out of reach with current knowledge and methods used for computational approaches.

Comprehensive mutational studies, including not only single variants other than Ala, but also multiple variants, are inescapable to unravel the relationship between structure and affinity [21]. Such studies are scarce [22–23]. They cannot be exhaustive due to the extremely large size of the mutational landscape. Furthermore an increase in library size is generally achieved at the expense of detailed activity quantifications. Illustrations of large-scale studies are provided by the combinatorial mutagenesis of the interface between the human growth hormone and its receptor [24], the mutational analysis of a PDZ domain in the cellular context [25] and the high resolution mapping of the interaction between the WW domain and a peptide using protein display combined with high-throughput sequencing [26]. While double mutant behavior could generally be predicted from that of single mutants in the latter study, the two former studies stress the context-dependent effect of some interface mutations.

Residue interdependences (defined here as those cases when a mutation at position A modifies the impact on binding affinity of a mutation at position B) surmise a modified role of the wild-type (WT) and replacing residues in the binding mechanism, such as the existence of different contact types or geometries. Here we search for residue interdependences within the peptidic epitope recognized by the single chain antibody fragment (scFv) 1F4 [27], previously proposed to be capable of different recognition modes [28]. ScFv1F4 binds with high affinity to peptides corresponding to the N-terminus of Human Papilloma Virus 16 oncoprotein E6 (HPV16-E6) [29]. In a previous work, a full permutation analysis of the WT decapeptide ^6^TAMFQDPQER^15^ allowed us to identify a well-defined epitope composed of positions 8 to 15, among which six showed a restricted tolerance to amino-acid changes and were considered as essential epitope residues [28]. Only the WT amino-acid or conservative changes were allowed at five of these essential positions (9–12 and 15). In contrast Phe and Gln, two amino-acids that differ in physicochemical properties, were allowed at position 13 while conservative replacements led to weaker binding signals. The observation that position 13 is essential for binding but can accommodate residues with a different bonding potential led us to propose that scFv1F4 uses alternative peptide binding mechanisms. If several residues are implicated in the alternative mechanisms, residue interdependences should be observed: the susceptibility of the binding affinity to amino-acid replacements at a given position should depend on the nature of the amino-acid present at another position. Such occurrences are readily identified from comparing the permutation patterns (effect on binding of replacing each position by all amino-acids) of variant peptides with that of the WT peptide. Here we present the permutation analysis of the WT peptide and of 23 starting variants performed using the PEPperPRINT GmbH facility (PEPperCHIP® Platform technology, Heidelberg, Germany). A subset of the peptide–scFv interactions was characterized by SPR (Biacore®, GE Healthcare Biacore, Uppsala, Sweden). Our data indicate that replacements at peptide positions 11, 12 or 13 modify the impact on the binding affinity of amino-acid changes at the other two positions, demonstrating a dependence between the three residues for scFv1F4 binding, and supporting the existence of alternative binding modes. Furthermore the alternative modes entailed high-affinity peptide recognition.

## Materials and Methods

Materials and methods were described in detail previously [28] and will be briefly summarized here.

### Soluble peptides and recombinant antibody fragments

ScFv1F4 [27] and scFv1F4-Q_L34_S (scFv1F4 with a Q34S replacement in the light chain [30]) were expressed and purified as described previously [28]. Soluble peptides derived from residues 6–15 (^6^TAMFQDPQER^15^) of oncoprotein E6 of human papilloma virus 16 (HPV16-E6) were purchased from ProteoGenix (Oberhausbergen, France). The variants will be denoted either by their sequence, with the modified position in bold, or by the replacement (for example ^6^TAMFQDP**F**ER^15^ or Q_13_F).

### Peptide array synthesis and analysis

**Fig 1:**
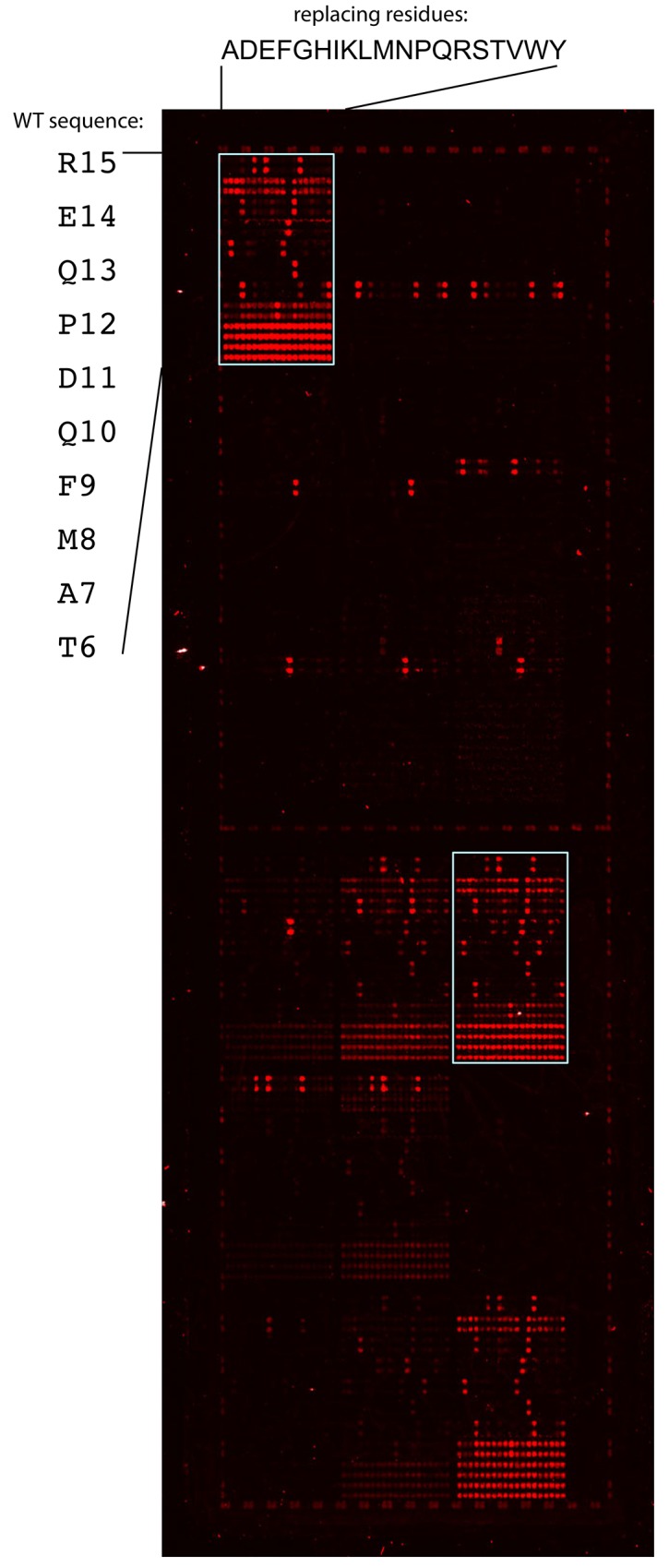
Stained PEPperCHIP® array B. The permutation scans of the WT peptide (upper left) and of variant ^6^TAMFQDP**F**ER^15^ are highlighted by white frames. Replaced positions together with the WT amino-acid are indicated on the left of the upper left frame. Amino-acids introduced at each position are indicated on top of the frame. Duplicated spots are easily identified.

ScFv1F4-Q_L34_S labeling with DyLight680, peptide array synthesis and binding detection were performed by PEPperPRINT GmbH (PEPperCHIP® Platform Technology, Heidelberg, Germany). The permutation scans were carried out on the WT decapeptide ^6^TAMFQDPQER^15^ and on its variants. In a permutation scan, the effect on binding of replacing each of the ten peptide positions by all amino-acids except cysteine is analyzed, which requires the synthesis of 190 spot peptides per starting peptide (19 amino-acids at 10 positions). Two arrays were prepared, each containing the permutation analysis of the WT peptide and of 19 variants. All peptides were synthesized in duplicate (7,600 peptide spots per array). The peptide arrays were stained with the DyLight680-labeled scFv1F4-Q_L34_S. The staining pattern of array B is shown in Fig 1. The permutation scans of the WT peptide and of variant ^6^TAMFQDP**F**ER^15^ are highlighted by white frames. The staining pattern of array A (S1 Fig) was published previously [28].

### SPR experiments

SPR kinetic experiments were carried out using a Biacore T200 instrument (GE-Healthcare Biacore, Uppsala, Sweden) as described previously [28]. Soluble peptides containing a C-terminal cysteine were covalently coupled to the sensor surfaces using the thiol coupling chemistry. The running buffers for kinetic measurements were HBS (HEPES buffered saline: 10 mM HEPES, 150 mM NaCl, 3.4 mM EDTA, pH 7.4) or HBS-200 (10 mM HEPES, 200 mM NaCl, pH 7.4), both supplemented with 0.005% (v/v) surfactant P20. Three-fold dilutions of a 45 or 30 nM scFv1F4 sample were injected over the surfaces in multiple cycle kinetic experiments. Injection and post-injection times were 180 s and 600 s, respectively. The flow rate was 40 μL/min. The double-referenced binding curves were fit to the one:one Langmuir binding model, using the Biacore T200 evaluation software (GE Healthcare Biacore).

### Circular dichroism experiments

Circular dichroism (CD) experiments were recorded on a Jasco J-815 spectropolarimeter (Easton, MD) equipped with an automatic 6- position Peltier thermostated cell holder. Samples (65 μL) at 400 μM were prepared in HBS buffer. Far-UV CD data were collected in the 190–270 nm range using a 0.1 mm pathlength cell (Quartz-Suprasil, Hellma UK Ltd) at 25.0°C ± 0.1°C. Spectra were acquired using a continuous scan rate of 100 nm/min and averaged over 10 successive scans. The response time and the bandwidth were 1.0 s and 1 nm, respectively. The solvent spectrum was subtracted from spectra obtained under identical conditions. Far UV data are deconvoluted with the CDPro package [31] using the CONTINLL algorithm with the SDP48 protein database, and means and standard deviations calculated over the results obtained for the validated structures.

### NMR spectroscopy

Samples for NMR spectroscopy were prepared in HBS buffer supplemented with 7% D2O. Spectra were acquired at 25°C on a Avance III 700 MHz spectrometer (Bruker) equipped with a Z-gradient triple resonance cryoprobe. Spectra were processed with NMRnotebook (NMRTEC, Illkirch, France).

## Results

### Peptide array design

The objective was to investigate whether a starting amino-acid replacement modifies the effect on binding of amino-acid changes at neighboring positions. For example the effect of a deleterious starting change could be compensated by a replacement elsewhere. Residue interdependences may also be found in the case of non-deleterious starting replacements. They can be identified by comparing the permutation patterns of the WT and variant peptides. Arrays A [28] (S1 Fig) and B (Fig 1) each contained the permutation scans of the WT decapeptide ^6^TAMFQDPQER^15^ and of nineteen variants. Data recorded for the WT and 14 variant peptides of array A were described previously [28]. The data presented here include all permutation scans in array B (WT and 19 variants), together with those of 5 variants in array A. One of these was performed twice in array A (peptide ^6^T**R**MFQD**V**QER^15^). Therefore the data include the permutations scans of the WT and of 23 variant decapeptides (Table 1), each of them being recorded at least in duplicate.

**Table 1:** Spot fluorescence data for the starting peptides.

Array	Sequence^a^	Nb spots	Mean Int.^b^	Standard deviation (at 1 σ) ^c^	% of WT signal
A	**TAMFQDPQER** ^**d**^	32	**6800**	1300	**100**
A	TRMFQDVQER	42	340	120	5
A	TAMFQDVFER	20	**940**	190	**14**
A	TAMFQSPYER	20	**1410**	430	**21**
A	TQMFQDPYER	22	**1580**	370	**23**
B	**TAMFQDPQER**	46	**18100**	2100	**100**
B	TAMDQDPQER	24	500	530	3
B	TAMLQDPQER	26	480	270	3
B	TAMFADPQER	26	430	200	2
B	TAMFKDPQER	24	390	200	2
B	TAMFQTPQER	22	580	400	3
B	TAMFQDQQER	28	800	1100	5
B	TAMFQDHQER	28	720	450	4
B	TAMFQDYQER	28	1280	750	7
B	**TAMFQDVQER**	30	**2900**	1000	**16**
B	**TAMFQDPYER**	26	**6800**	1300	**37**
B	**TAMFQDPFER**	24	**12300**	1300	**68**
B	TAMFQDPQEA	24	1060	210	6
B	**TAMFQDPQEQ**	24	**4330**	390	**24**
B	TAMLADPQER	24	510	340	3
B	TAMFQRAQER	20	470	360	3
B	**TAMFQDVYER**	24	**2940**	670	**16**
B	**TGSFQDPQER**	20	**11900**	1500	**66**
B	TAMFQDPSFM	20	420	370	2
B	TGSFQRASFM	20	430	140	2

^a^ Sequence replacements relative to WT are in red.

^b^ Mean intensities > 10% that of the WT peptide are in bold.

^c^ Standard deviation calculated as $s=\sqrt{\frac{1}{N-1}\sum_{i=1}^{N}{\left(x_{i}-\bar{x}\right)}^{2}}$

^d^ The permutation of the WT decapeptide in array A was described in Vernet *et al*., 2015 [28].

A search for interdependences between all possible residue pairs is unfeasible. We therefore focused on the consequences on the permutation patterns of replacing essential residues. Thirteen variants presented a single change at one of the six essential positions (9 to 13 and 15), nine presented multiple changes including at least one at an essential position, and one was replaced at non-essential positions (A_7_G-M_8_S). The effect on binding of the replacements ranged from minor to drastic, as emphasized when the averaged fluorescence signals are expressed as percentage of that recorded with the WT decapeptide (column 6 in Table 1).

### Array data analysis

The repeatability of fluorescence measurements can be assessed because: *i/* all peptides were synthesized in duplicate (Fig 1), *ii/* the duplicated scan of peptide ^6^T**R**MFQD**V**QER^15^ was repeated in array A and *iii/* several peptides occur repeatedly within a same or in different families. Array B contained 3,222 different peptides, with 105 peptides repeated between 6 and 46 times. The standard deviation on the mean intensity calculated for these repetitions was < 2,100 (Table 1), which is 12% of the WT intensity reading (18,100), demonstrating an excellent signal repeatability, as was also observed for array A [28].

**Fig 2:**
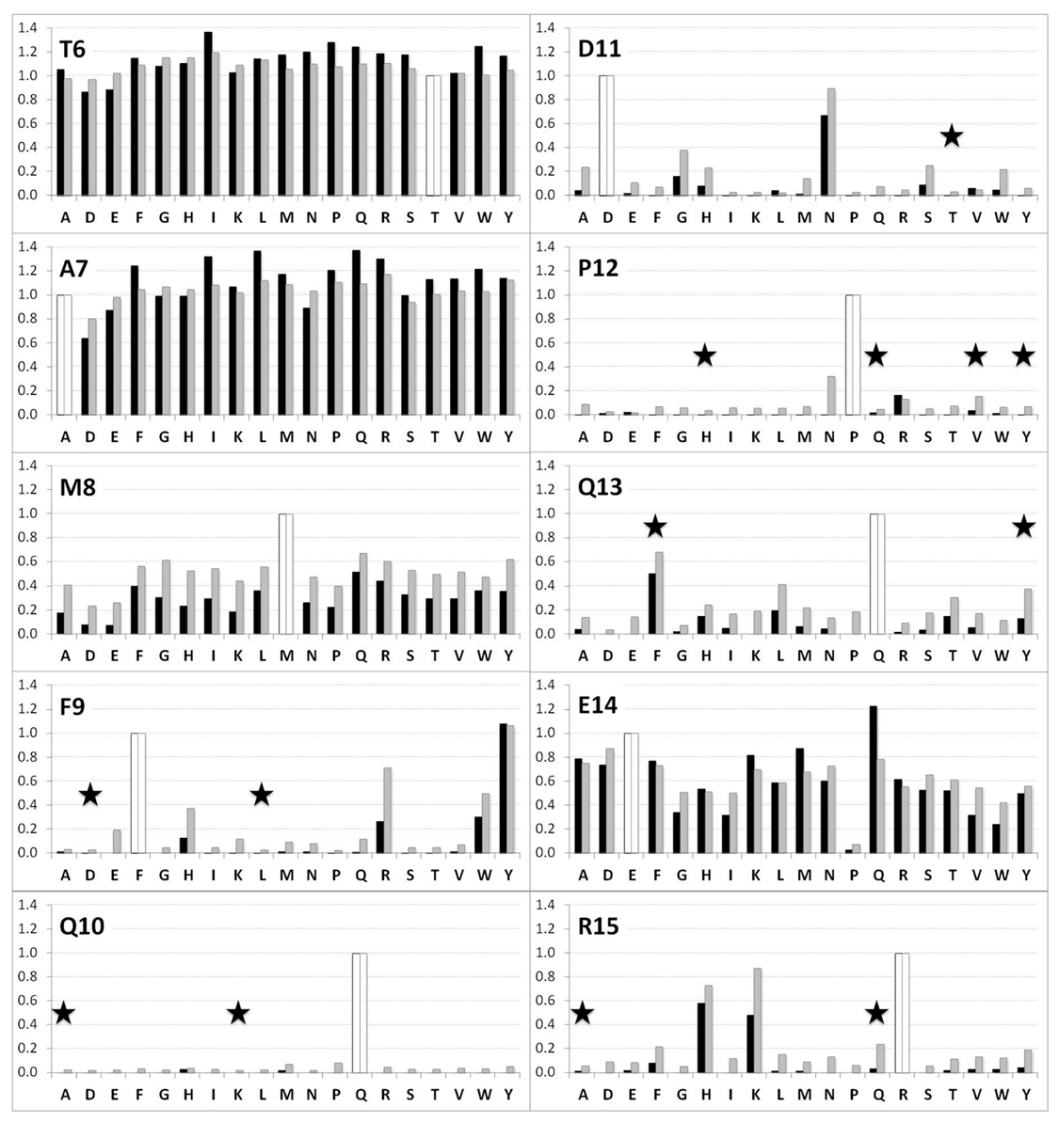
Comparison of the two WT permutation patterns. Intensities were normalized with respect to the mean intensity recorded for the scFv1F4—WT peptide interaction. Black and grey bars correspond to the normalized intensities in arrays A and B, respectively. White bars represent the WT sequence (normalized value = 1). Stars indicate single replacements in starting variants (see Table 1).

The comparison of the WT permutation scans, performed in both arrays A and B, further demonstrated data quality. The signal intensity ranges differed in the two arrays: 6,800 and 18,100 for the scFv—WT decapeptide interaction in arrays A and B, respectively (Table 1). However normalization of the intensity values with respect to the mean value of the scFv1F4-WT peptide intensity in each panel, produced well-superimposed patterns (Fig 2). The data confirm the conclusions that positions 9 to 13 and 15 show a restricted tolerance to amino-acid changes (F_9_, Y_9_—Q_10_—D_11_, N_11_—P_12_—Q_13_, F_13_ and H_15_, K_15_, R_15_) and represent essential epitope positions [28]. In addition, assuming that T6 is not part of the epitope, the variability observed for this position (standard deviation = 0.12) provides insight into the statistical significance of the normalized intensities.

### Comparison of WT and variant permutation patterns

The averaged fluorescence signals recorded for fourteen starting peptides were below 10% of that recorded with the WT peptide (Table 1). These peptides carried one or several replacements at essential positions 9, 10, 11, 12 and 15. In contrast, four single replacements (P_12_V, Q_13_Y, Q_13_F and R_15_Q) had a milder effect with signals at 16, 37, 68 and 24% of the WT signal, respectively. Furthermore five double variants showed signals that exceeded 10% of the WT signal. Four of these included the Q_13_F or Q_13_Y change (P_12_V-Q_13_Y, P_12_V-Q_13_F, D_11_S-Q_13_Y, A_8_Q-Q_13_Y) and one was modified at non-essential positions 7 and 8 (A_7_G-M_8_S).

**Fig 3:**
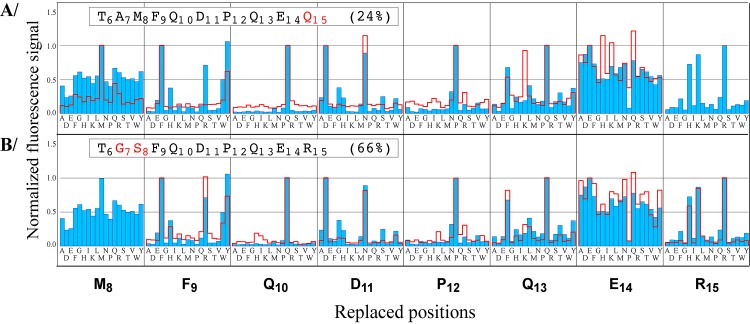
WT-like permutation patterns (positions 8–15). The patterns of variants R_15_Q (A) and A_7_G-M_8_S (B) are represented as red lines and superimposed with the WT pattern (blue). Fluorescence signals were normalized with respect to that recorded with each starting peptide. Patterns at replaced positions are not shown because they are not relevant to the modified sequence context.

**Fig 4:**
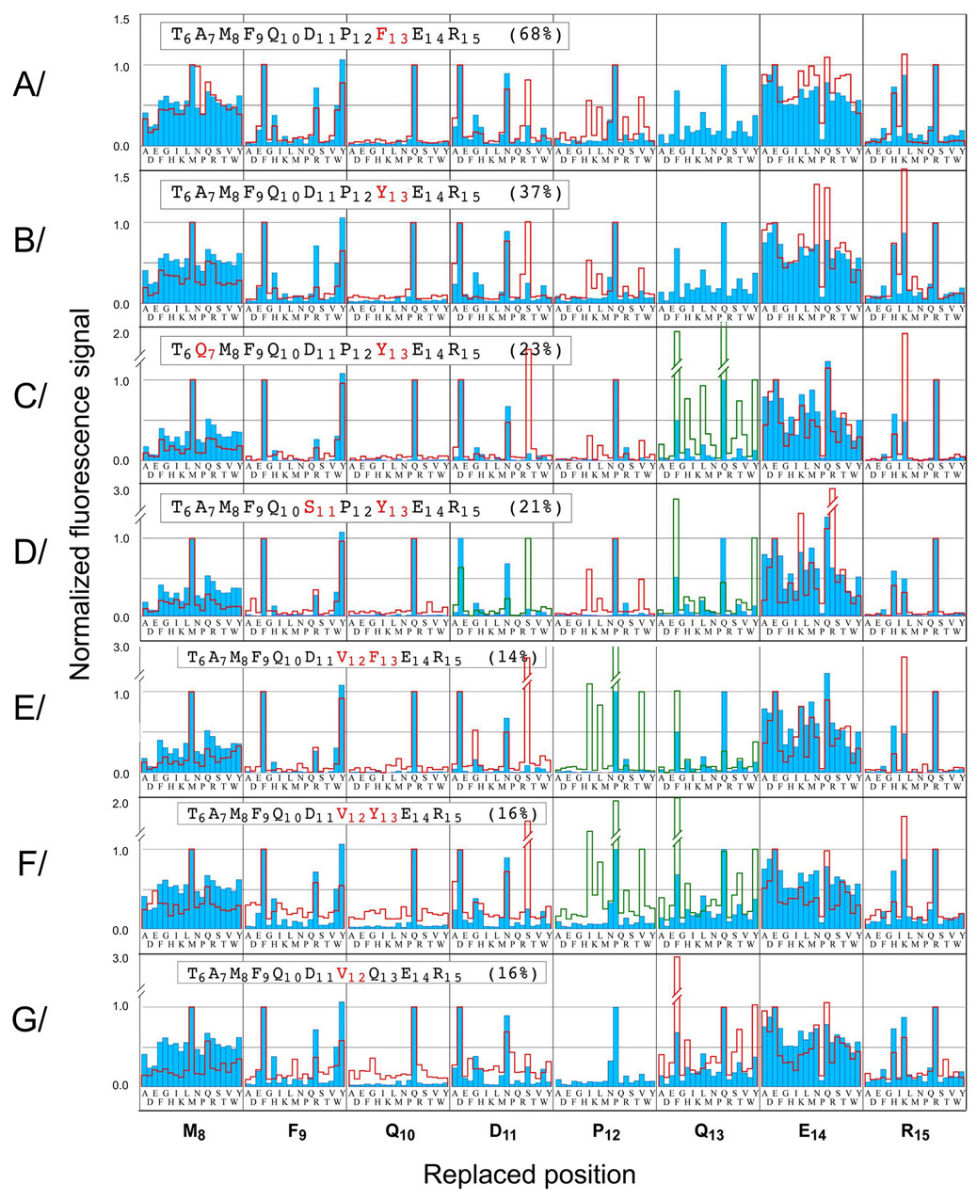
Permutation patterns for variants modified at positions 11, 12 and 13. Variant patterns are represented as red lines and superimposed with the WT pattern (blue). Fluorescence signals were normalized with respect to that recorded with each starting peptide. Patterns at the replaced positions are in green for double variants and not shown for single variants.

In order to compare the permutation patterns of the variants with that of the WT, the mean fluorescence signals were normalized as % of the signal recorded with each starting peptide. Figs 3, 4 and S2 Fig show the superimpositions of the normalized WT (blue) and variant (red line) patterns. Positions 6 and 7 are not represented because they were tolerant to all replacements in all peptides analyzed. The replacement patterns at positions modified in the starting sequence are not relevant to the modified sequence context. Therefore they are not represented in single variants, and represented in green in multiple variants. For the cluster of the fourteen peptides displaying strongest effect upon mutation (averaged fluorescence signals below 10% of the WT signal), scFv1F4 binding could not be restored by non-WT replacements, as illustrated by the permutation patterns of four of the fourteen peptides (S2 Fig). Among the nine variant binders displaying milder effect, two (A_7_G-M_8_S, R_15_Q) showed WT-like patterns (Fig 3). In contrast the permutation patterns of positions 11 and 12 were modified in variants presenting the Q_13_Y or Q_13_F replacements. Indeed, in the context of these starting peptides, S at position 11 was consistently compatible with binding in addition to D_11_ and N_11_ (Fig 4A–4F), while I, L and V were allowed at position 12 in addition to the WT residue P_12_ (Fig 4A–4D). Concomitantly, F_13_ was preferred over the WT residue Q_13_ in the context of starting peptides containing the P_12_V mutation (Fig 4G) or the D_11_S mutation (green traces in Fig 4D). Data from seven distinct permutation scans in two different arrays were thus fully consistent. They indicate interdependence between positions 11, 12 and 13.

### SPR validation

**Fig 5:**
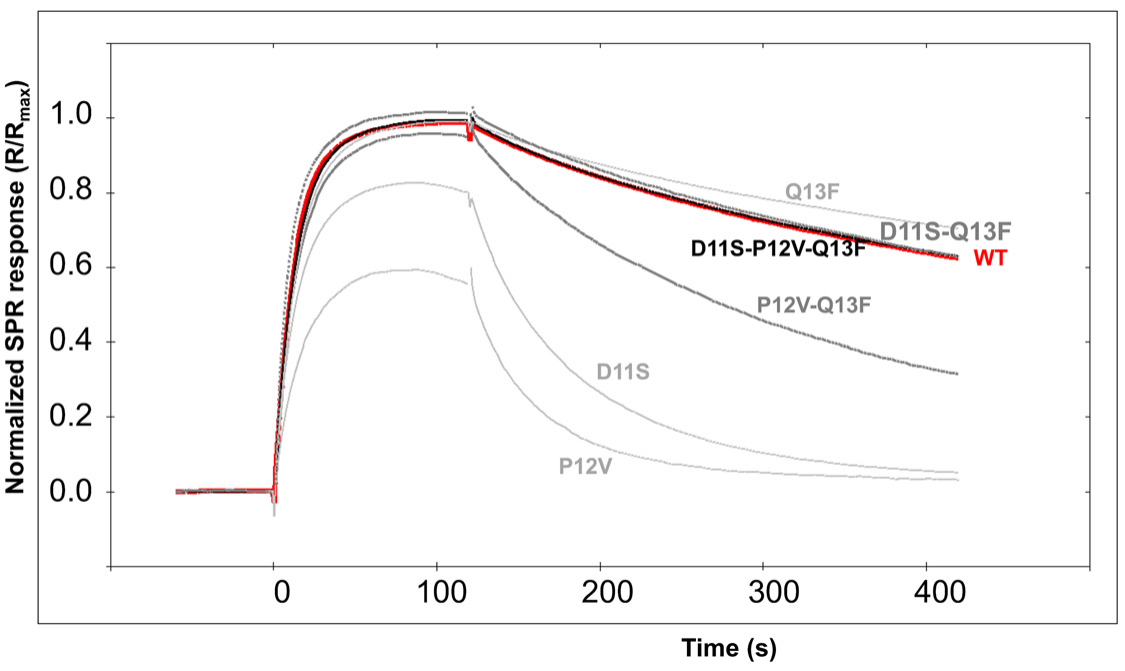
Normalized SPR kinetic curves. The purified scFv1F4 (10 nM in HBS) was injected over peptide surfaces. The WT curve is shown in red, single variants in light grey, double variants in dark grey and the triple variant in black. The curves were superimposed using the software TraceDrawer (Ridgeview Instruments AB, Uppsala, Sweden).

**Fig 6:**
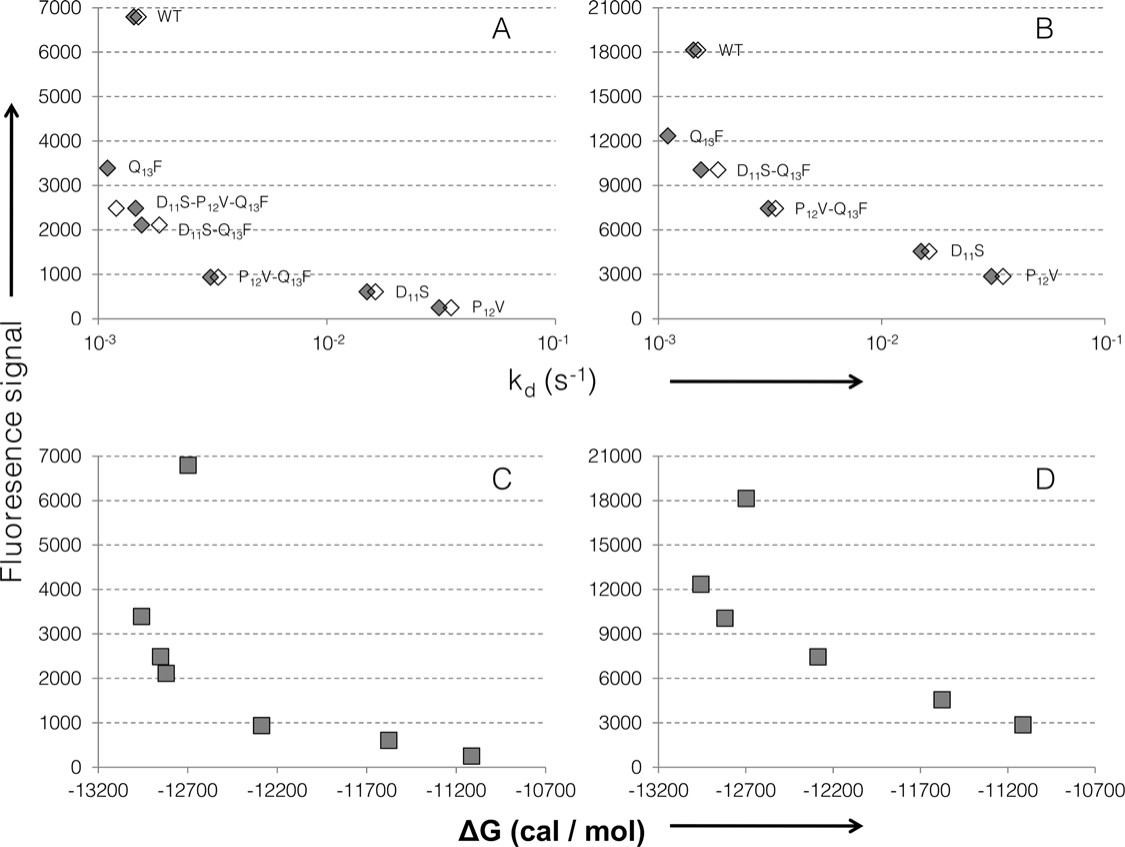
Correlation between SPR constants and fluorescence signals. The k_d_ (A, B) and ΔG (C, D) values are plotted against fluorescence signals from arrays A (A,C) and B (B,D). The k_d_ as measured in HBS (grey markers) and HBS-200 (white markers) is shown in log scale. Peptide names are indicated in A and B.

Eight peptides were synthesized for SPR characterization of their interaction with scFv1F4 (Table 2). The variant P_12_Y was used as a control of weak binding, its fluorescence signal being < 10% that of the WT (Fig 2). The seven other peptides were designed to investigate the interdependence between positions 11, 12 and 13 by comparing the effect on binding of a given replacement in various sequence contexts. Fig 5 shows the normalized kinetic curves (signal expressed as % of the maximal binding capacity of the surface) recorded when injecting a 10 nM purified scFv1F4 sample in HBS over surfaces carrying these seven peptides. The kinetic and affinity constants are listed in Table 2, together with ΔG (cal / mol) calculated as RT x log K_D_. Association rates were similar for all complexes (k_a_ in the range 1 to 5 x 10^6^ M^-1^ s^-1^), but dissociation rate constants differed significantly. The k_d_ values measured in HBS and HBS-200 (HBS containing 200 instead of 150 mM NaCl) were similar (S1 Table) and correlated well with the fluorescence signals measured in the spot approach (Fig 6A and 6B). The outlier is the WT peptide. The correlation between binding free energies (in HBS) and fluorescence signals is shown in Fig 6C and 6D.

**Table 2:** Binding parameters deduced from SPR measurements in HBS.

Peptide ^a^	k_a_ (10^6^ M^-1^ s^-1^)	k_d_ (10^−3^ s^-1^)	K_D_ (10^−9^ M)	ΔG (kcal/mol)	Number of experiments
TAMFQDPQERC	3.05 ± 0.9	1.43 ± 0.05	0.47 ± 0.15	-12.7 ± 0.2	6
TAMFQDP**F**ERC	3.75 ± 1.15	1.10 ± 0.01	0.29 ± 0.09	-13.0 ± 0.2	2
TAMFQ**S**PQERC	4.70	15.00	3.20	-11.6	1
TAMFQD**Y**QERC	1.63 ± 0.78	133 ± 47	82 ± 68	-9.2 ± 0.5	3
TAMFQD**V**QERC	4.20	31.00	7.40	-11.1	1
TAMFQD**VF**ERC	3.36 ± 1.08	3.10 ± 0.71	0.92 ± 0.51	-12.3 ± 0.3	7
TAMFQ**SVF**ERC	4.26 ± 1.35	1.46 ± 0.05	0.34 ± 0.12	-12.9 ± 0.2	5
TAMFQ**S**P**F**ERC	4.85 ± 2.35	1.55 ± 0.05	0.32 ± 0.17	-13.0 ± 0.3	2

^a^ Sequence replacements relative to WT are in bold and underlined.

### Structural characterization of the peptides

**Fig 7:**
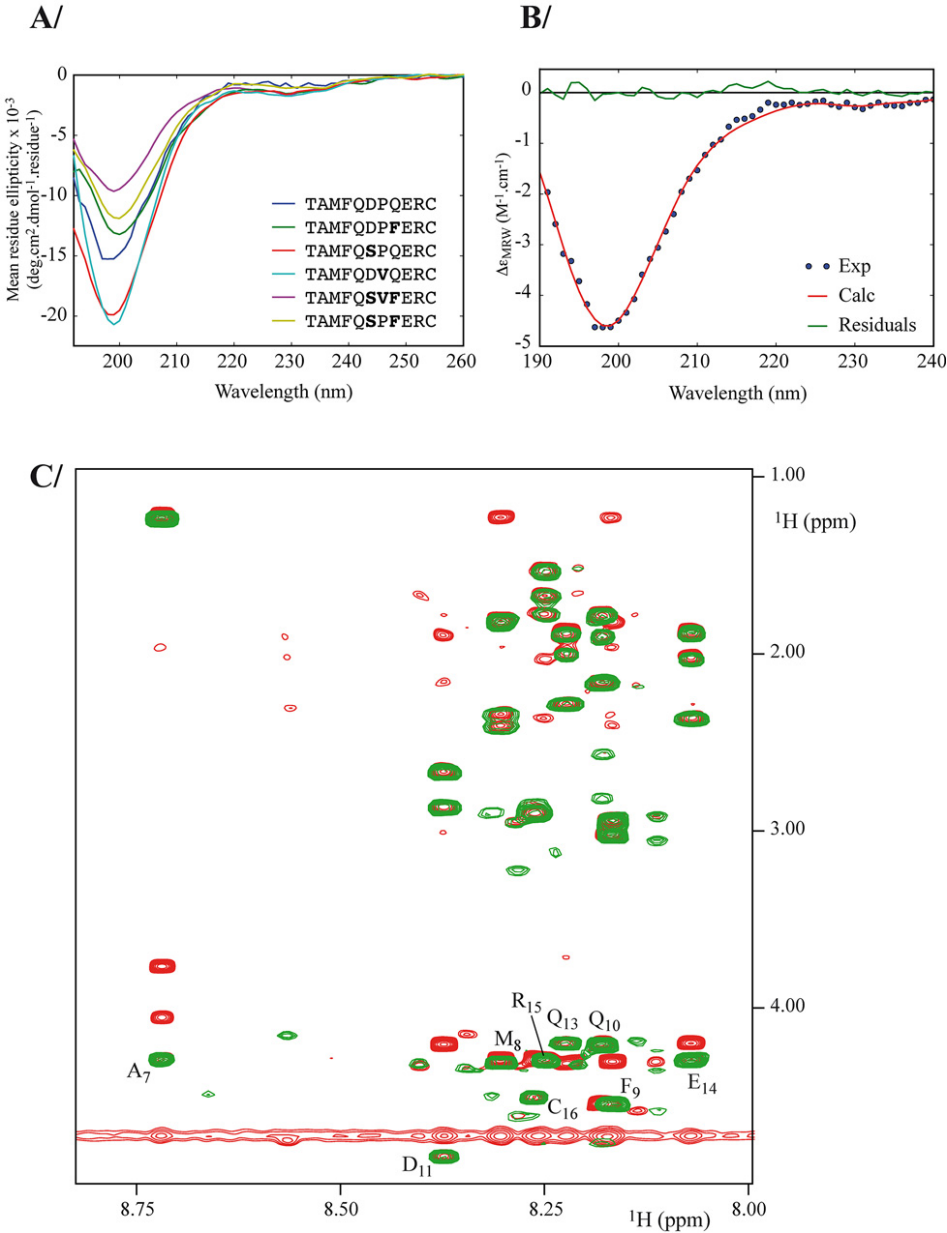
Structural characterization of a subset of peptides. A) CD spectra of 6 of the peptides listed in Table 2. The CD signal is expressed as mean residue ellipticity. B) Representative analysis of far UV data deconvoluted with the CDPro package using the CONTINLL algorithm with the SDP48 protein database (See S2 Table for details). The CD signal is expressed as mean residue CD extinction coefficient. C) Superimposition of NMR 1H-1H TOCSY (green) and NOESY (red) spectra recorded at 700 MHz on a 1 mM sample of WT peptide. All spectra were measured at 25°C in HBS buffer. Sequential assignment is reported here according to the peptide numbering. Note that P12 is missing due to the lack of NH in the proline residue.

To investigate if differences in dissociation rate constants are related to structural changes in the free peptide, far-UV circular dichroism (CD) spectra were recorded for a subset of peptides (Fig 7A). The CD spectra present profiles that are typical of disordered structures. This observation is confirmed by deconvolution of the CD spectra indicating a very low prevalence of alpha and beta secondary structure content (always below 25%) (Fig 7B and S2 Table). These results imply that the mutations, even involving the proline residue at position 12, did not induce drastic changes in the peptide conformation as the proportion of secondary structure elements stayed rather constant. To better characterize the conformation of the free WT peptide in solution, NMR experiments were also conducted (Fig 7C). Both 1H-NMR TOCSY and NOESY spectra exhibit a rather narrow amide proton chemical shift dispersion limited to 0.7 ppm. Such a range is characteristic of a lack of structural organization of the peptide backbone as well. Note that region 8 to 15 in the NMR structure of the free N-terminal domain of E6 is highly flexible (PDB ID 2LJX [32]).

## Discussion

The analysis of peptide–protein interactions is of general relevance as a number of interactions that mediate key cellular processes involve peptides or short segments present in disordered protein regions or in protein loops [33]. Furthermore bioactive peptides, or peptides able to interfere with protein-protein interactions, are actively developed for biomedical applications [34,35]. In particular peptides are developed as vaccines [36] and their antibody binding modes have been investigated in structural or functional studies [37]. Multiple binding modes attributed to peptide or paratope plasticity have been observed in the case of weak-affinity complexes formed by peptides with anti-protein antibodies or primary repertoire antibodies [38–41]. Affinity and selectivity have been improved by constraining the peptides [42–45]. In contrast, we describe here short linear peptides, likely to be largely unstructured in solution according to CD and NMR experiments (Fig 7), that display < nM antibody binding affinity.

We demonstrate that scFv1F4 recognizes with strong affinity peptides that possess alternative three-residue motifs at essential positions 11 to 13 (WT motif ^11^DPQ^13^, variant motifs ^11^SVF^13^ and ^11^SPF^13^), within a common motif (F_9_—Q_10_ and R_15_). Moreover the large-scale mutational study also provided information on non-binders, demonstrating that the antibody fragment is selective. Information on non-binders is generally scarce due to the technical challenges of producing large numbers or variants and of quantifying their binding properties. Nevertheless it is essential as the ability of a protein to bind its partner(s) is as important as its inability to interact with non-wanted homologues to ensure correct biological function.

### Ala-replacements of the WT peptide identify six hot-warm spots

We define hot-warm spots as residues whose Ala replacement results in an increase in binding free energy > 1.5 kcal/mol. The ΔG of the WT peptide–scFv1F4 interaction, as deduced from SPR kinetic constants, was -12.7 kcal/mol (K_D_ = 0.5 nM, Table 2). Interactions with ΔG > -11.2 kcal/mol (K_D_ > 6 nM) thus display an increase in binding free energy > 1.5 kcal/mol. From the relation between fluorescence intensities and ΔG in HBS (Fig 6C and 6D), we can roughly approximate intensities < 250 in array A or < 3000 in array B with ΔG > -11.2 kcal/mol. Four Ala variants showed fluorescence readings below these limits: F_9_A, Q_10_A, P_12_A and R_15_A (signals of 100, 54, 84 and 86, respectively, in array A, and of 633, 425, 1644 and 1057, respectively, in array B). These observations are consistent with the ΔΔG > 2 kcal/mol previously measured by SPR for the P_12_A (2.7 kcal/mol) and Q_13_A (2.4 kcal/mol) replacements [29]. Variants Q_13_A and D_11_A showed intensities slightly above these limits (intensities of 252 and 305, respectively, in array A, and of 2524 and 4298, respectively, in array B). In conclusion the Ala replacement data, together with the restricted tolerance to amino-acid changes at six positions (Fig 2), point to the strong peptide recognition selectivity of scFv1F4.

### Substitutional analysis of variants identifies both independent and interdependent effects of replacements

**Fig 8:**
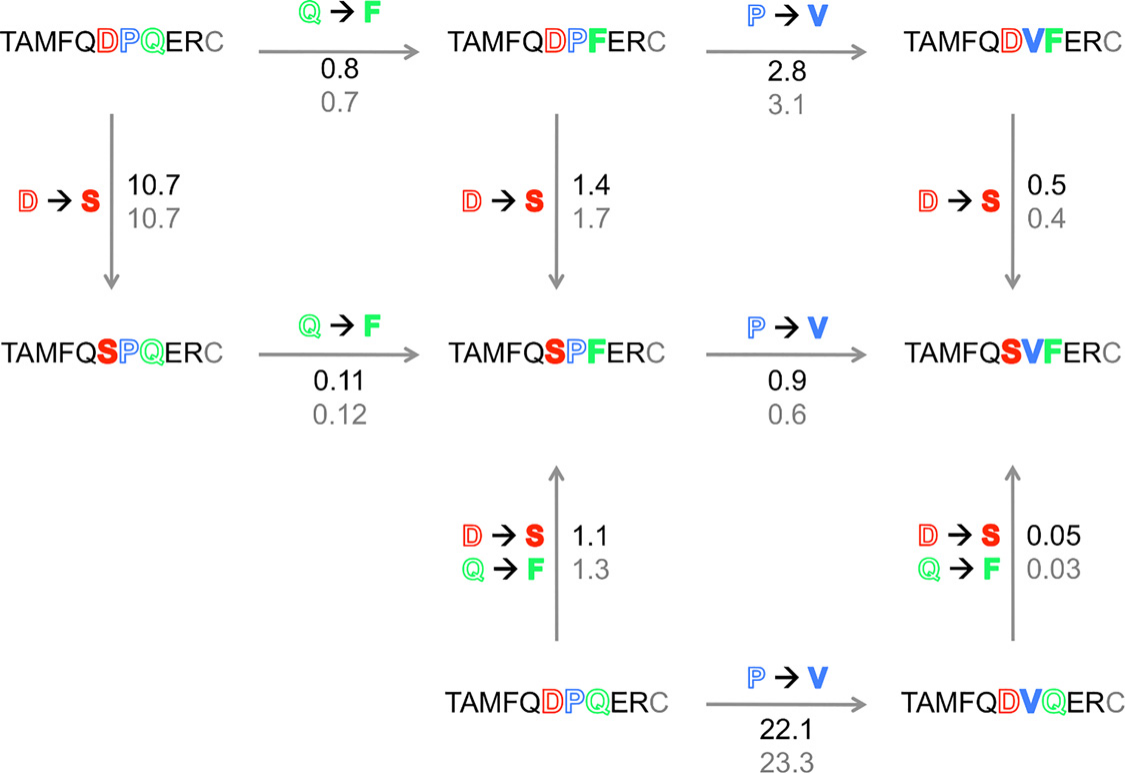
Context dependence of the effect on k_d_ of replacements. Positions 11, 12 and 13 in the peptide sequences are shown in red, blue and green, respectively. The filling is white for the WT residue (D_11_, P_12_, Q_13_) and colored for the modified residue (S_11_, V_12_, F_13_). The ratio k_d_ variant / k_d_ WT, as measured by SPR in HBS (black) or HBS200 (grey), is given next to each arrow, together with the nature of the replacement.

Residue interdependences were detected within the set of positions 11–13, but not elsewhere. For example the replacement R_15_Q decreased fluorescence intensity (24% of the WT intensity, Table 1) but the permutation pattern of positions 6 to 14 was nearly unaffected (Fig 3B). The conserved tolerance to amino-acid changes in variant R_15_Q, despite a decreased fluorescence signal compared to the WT (Table 1), indicates independent mutational effects: the starting change does not influence the effect of replacements at other peptide positions. The R_15_Q replacement is likely to modify the scFv-peptide interface only locally. In contrast a replacement at one of the hot-warm spots 11–13 affected the tolerance to changes at the two other positions (Fig 4), as also confirmed by SPR (Figs 5 and 6). This finding supports the hypothesis that scFv1F4 is capable of distinct peptide binding modes, initially proposed on the basis that Q and F, two amino-acids with different physico-chemical properties, are equally well tolerated at position 13 [28]. Fig 8 compares the effect on k_d_ of a given replacement in peptides with different sequences. The D_11_S replacement (top vertical arrows) increased k_d_ 11-fold in the WT context, but had only a marginal effect in ^6^TAMFQDP**F**ER^15^ and ^6^TAMFQD**VF**ER^15^. Similarly the P_12_V replacement (right-hand horizontal arrows) increased k_d_ 22 to 23-fold in the WT peptide, but had a weak or no effect in ^6^TAMFQDP**F**ER^15^ and ^6^TAMFQ**S**P**F**ER^15^. These effects can also be visualized in Fig 5 by comparing the dissociation profiles obtained for each peptide.

A mutational study of the interaction between TEM1-lactamase and lactamase inhibitor protein (BLIP) suggested a modular architecture of protein–protein binding sites, with mutations showing cooperativity within modules and additivity across modules [12]. The existence of other interdependences within the peptide recognized by scFv1F4, involving replacements that were not investigated, or interactions with stability below the detection limit of the spot experiments, cannot be excluded. Nevertheless the data recorded so far suggest that they are restricted to three of the six essential epitope positions.

### ScFv1F4 recognizes alternative motifs with strong affinity

The multiple variants D_11_S-Q_13_F and D_11_S-P_12_V-Q_13_F displayed stronger scFv binding compared to single variants (D_11_S or P_12_V) in SPR experiments, illustrating a compensating effect of amino-acid changes and confirming the conclusions of spot experiments. The latter clearly indicated a greater tolerance to S at position 11 and I, L or V at position 12 in variants containing F_13_ instead of the WT Q_13_ (Fig 4). The ability of the scFv to recognize with similar affinity peptides that contain ^11^SVF^13^ and ^11^DPQ^13^ is corroborated with CD data showing almost no difference in structural contents. Even if the conformational ensembles are similar, the binding interfaces formed by the two bound peptides should differ due to the distinct nature of their side chains. Differences can be accommodated for instance if these residues are partially accessible to the solvent in the complex, or if the antibody binding site presents some plasticity [46–48]. However the recognition of peptides by the scFv is not ‘fuzzy’, as previously suggested [29] based on the observation that various sequences were compatible with binding activity in spot and phage display experiments. In the present work we demonstrate that the sequence requirements for high-affinity binding are restricted to a limited set of alternative amino-acid combinations: the D_11_S and P_12_V replacements required the concomitant Q_13_F change for optimal binding. ScFv1F4 is not a natural antibody, but a hybrid fragment formed from the light and heavy chain variable regions of two different monoclonal antibodies. Nevertheless the finding that alternative binding modes display sub-nanomolar affinity (Fig 5, Table 2) challenges the view that antibody selectivity is based on the existence of a unique optimal interface.

In a previous study we have shown that the “non-WT” binding motif was the only one selected from a phage-displayed peptide library [29]. The consensus of 20 clones was S_11_-P_12_-FY_13_. Seven clones contained ST-P-FY, 5 contained ST-VIL-FY and one D-P-F, in accordance with the present finding that these motifs lead to strong scFv1F4 binding. None of the phage-display sequences carried Q_13_. This selection bias cannot be explained by differences in binding properties as measured by SPR. It could originate from a bias in library composition or from differences in dynamics, which could influence preferential recognition in a competition situation.

The alternative motifs illustrate a case of concerted replacements that ensure optimal activity. Concerted (co-ordinated, co-evolving) amino-acid pairs, identified from sequence alignments of homologous proteins, were found to be closer together in space than random pairs [49,50]. Co-evolving information was then exploited to predict amino-acid structural contacts within protein cores, at protein interfaces and across interfaces [51–54]. The particular case described here, although not dealing with protein evolution, illustrates how individual changes (D_11_S, P_12_V) can be deleterious or not depending on a silent replacement (Q_13_F). Silent replacements would not necessarily co-evolve with other positions of a cluster, which might explain inconsistencies in the experimental validation of computational results [55].

## Conclusion

The relationship between affinity and selectivity is a matter of debate [56], high affinity being considered in some cases as a condition for specific binding and, in others, as favoring cross-reactivity. Numerous protein binders, and in particular antibodies, have been shown to form stable complexes with two or more ligands, but the assessment of selectivity requires the identification of a significant number of non-binders. Quantitative large-scale permutation studies represent a powerful tool to decipher the subtleties of protein-protein interactions, which cannot be inferred from mere Ala-scan experiments. Our results stress the importance and unpredictable outcome of residue dependencies in binding. We describe a high-affinity binder that is simultaneously selective (restricted tolerance to ligand changes) and permissive towards particular combinations of changes. It is not known if this dual behavior, identified for the interaction between an antibody fragment and a peptide, is an exception or not because few interaction systems have been studied at this level of quantitative detail.

## Supporting Information

S1 Fig Stained PEPperCHIP array A (adapted from [28] with permission). (TIF)

S2 Fig Permutation patterns of four non-binders. (TIF)

S1 Table Dissociation rate constants in HBS and HBS-200, as measured by SPR for 8 peptide-scFv1F4 interactions. (DOCX)

S2 Table Estimation of secondary structure elements of six peptides from experimental CD spectra. (DOCX)
