# Supplementary figures and images for: Antibody Binding Selectivity: Alternative Sets of Antigen Residues Entail High-Affinity Recognition
Source: PLoS One. 2015 Dec 2;10(12):e0143374. doi: 10.1371/journal.pone.0143374 (PMC4667898; doi:10.1371/journal.pone.0143374)

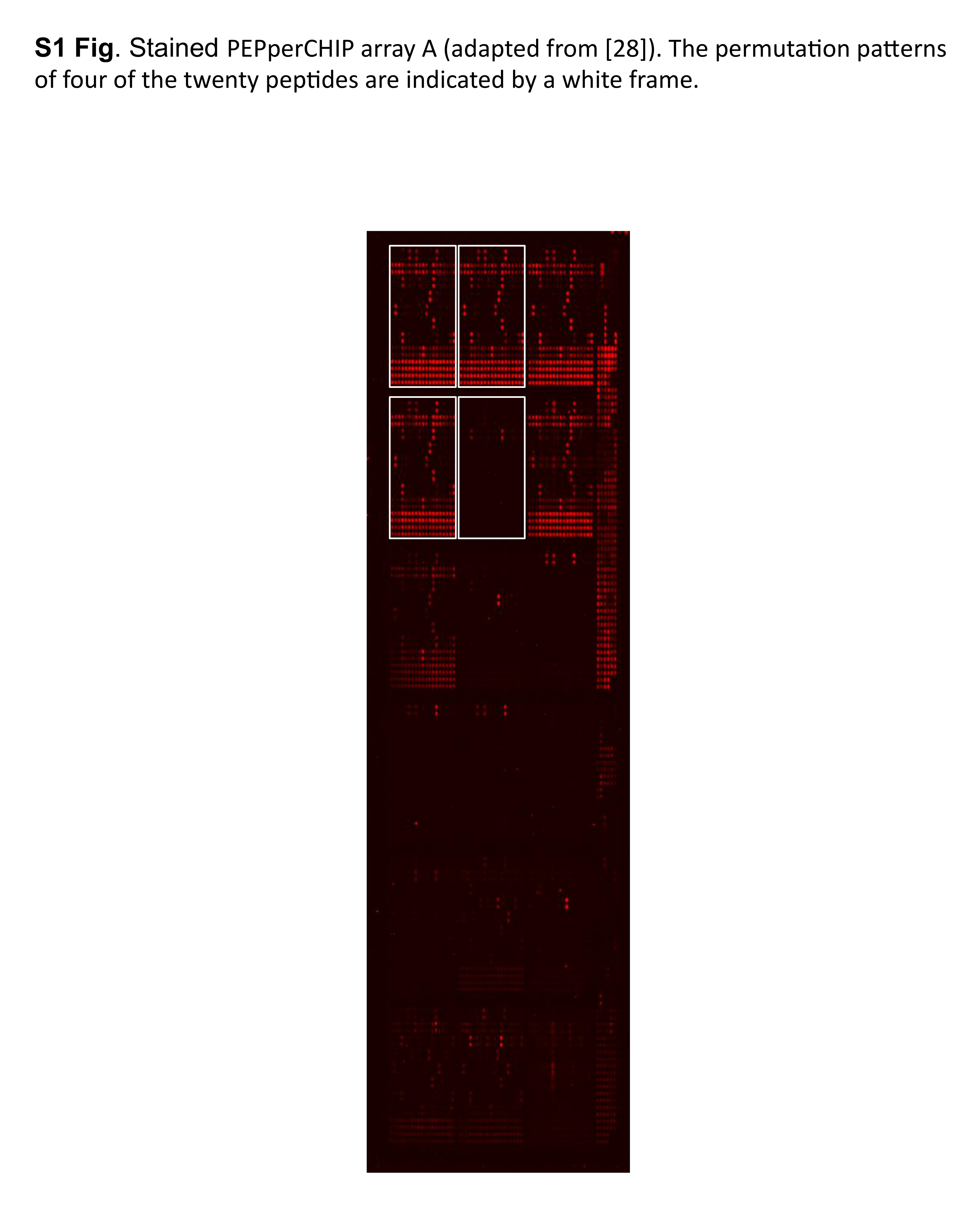

Supplement: S1 Fig — (TIF) [file pone.0143374.s001.tif]

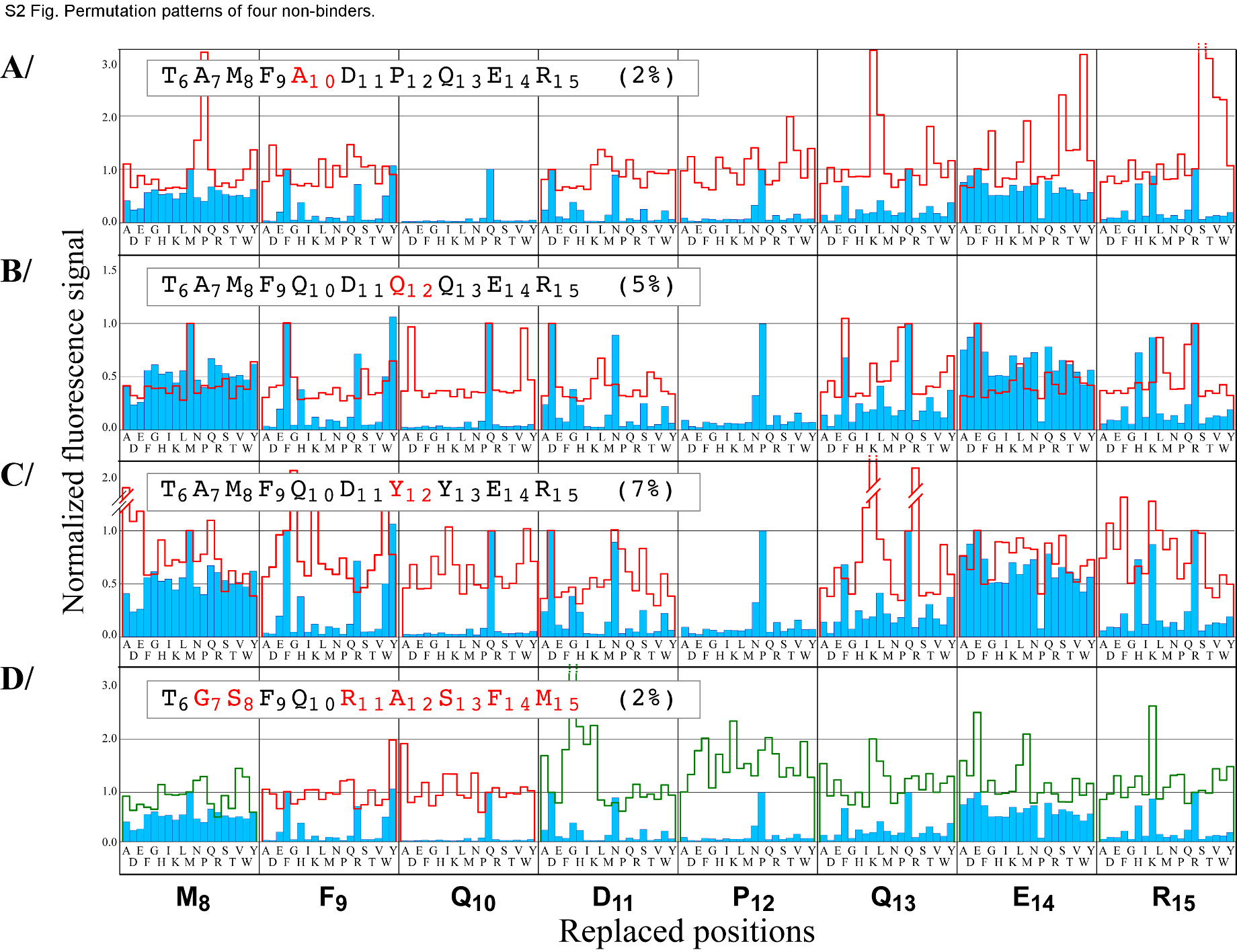

Supplement: S2 Fig — (TIF) [file pone.0143374.s002.tif]
