# Supplementary material for: Antibody Binding Selectivity: Alternative Sets of Antigen Residues Entail High-Affinity Recognition
Source: PLoS One. 2015 Dec 2;10(12):e0143374. doi: 10.1371/journal.pone.0143374 (PMC4667898; doi:10.1371/journal.pone.0143374)
Supplement: S1 Table — (DOCX) [file pone.0143374.s003.docx]

**S1 Table.** Dissociation rate constants in HBS and HBS-200, as measured by SPR for 8 peptide-scFv1F4 interactions

| **Peptide** | **Buffer** | **k_off_**  **(s^-1^ x 10^3^)** | **Number**  **experiments** |
| --- | --- | --- | --- |
| TAMFQDPQERC | HBS | 1.43 ± 0.05 | 6 |
|  | HBS-200 | 1.50 | 1 |
| TAMFQDP**F**ERC | HBS | 1.10 ± 0.00 | 2 |
|  | HBS-200 | 1.10 | 1 |
| TAMFQ**S**PQERC | HBS | 15.00 | 1 |
|  | HBS-200 | 16.33 ± 3.86 | 3 |
| TAMFQD**Y**QERC | HBS | 133.00 ± 47.00 | 3 |
|  | HBS-200 | 160.00 | 1 |
| TAMFQD**V**QERC | HBS | 31.00 | 1 |
|  | HBS-200 | 35.00 ± 15.00 | 2 |
| TAMFQD**VF**ERC | HBS | 3.10 ± 0.71 | 7 |
|  | HBS-200 | 3.35 ± 0.34 | 4 |
| TAMFQ**SVF**ERC | HBS | 1.46 ± 0.05 | 5 |
|  | HBS-200 | 1.20 ± 0.08 | 3 |
| TAMFQ**S**P**F**ERC | HBS | 1.55 ± 0.05 | 2 |
|  | HBS-200 | 1.85 ± 0.35 | 2 |
