# Supplementary material for: Antibody Binding Selectivity: Alternative Sets of Antigen Residues Entail High-Affinity Recognition
Source: PLoS One. 2015 Dec 2;10(12):e0143374. doi: 10.1371/journal.pone.0143374 (PMC4667898; doi:10.1371/journal.pone.0143374)
Supplement: S2 Table — (DOCX) [file pone.0143374.s004.docx]

**S2 Table.** Estimation of secondary structure elements of six peptides from experimental CD spectra. The values represent the means and standard deviations of the secondary structure results obtained from validated structures using the CONTINLL algorithm in combination with the SDP48 protein database in the CDPro package.

| Peptides | NRMSD | Alpha-helix (%) | Beta-strand (%) | Others (%) |
| --- | --- | --- | --- | --- |
| TAMFQDPQERC | 0.040 | 0.04 ± 0.02 | 0.21 ± 0.04 | 0.76 ± 0.06 |
| TAMFQDP**F**ERC | 0.059 | 0.04 ± 0.02 | 0.17 ± 0.04 | 0.79 ± 0.07 |
| TAMFQ**S**PQERC | 0.027 | 0.03 ± 0.02 | 0.10 ± 0.03 | 0.86 ± 0.05 |
| TAMFQD**V**QERC | 0.025 | 0.03 ± 0.02 | 0.13 ± 0.05 | 0.84 ± 0.11 |
| TAMFQ**SVF**ERC | 0.057 | 0.03 ± 0.02 | 0.25 ± 0.04 | 0.72 ± 0.06 |
| TAMFQ**S**P**F**ERC | 0.053 | 0.03 ± 0.02 | 0.24 ± 0.05 | 0.73 ± 0.06 |
